# Longitudinal clusters of pain and stiffness in polymyalgia rheumatica: 2-year results from the PMR Cohort Study

**DOI:** 10.1093/rheumatology/kez533

**Published:** 2019-11-19

**Authors:** Sara Muller, Rebecca Whittle, Samantha L Hider, John Belcher, Toby Helliwell, Chris Morton, Emily Hughes, Sarah A Lawton, Christian D Mallen

**Affiliations:** 1 Primary Care Centre Versus Arthritis, School for Primary, Community and Social Care, Keele University, Keele, UK; 2 Haywood Academic Rheumatology Centre, Midlands Partnership Foundation Trust, Stoke-on-Trent, UK; 3 Keele Clinical Trials Unit, School for Primary, Community and Social Care, Keele University, Keele, UK; 4 Education Research Centre, Wythenshawe Hospital, Manchester University NHS Foundation Trust, Manchester, UK

**Keywords:** polymyalgia rheumatica, pain, stiffness, clusters, prognosis

## Abstract

**Objectives:**

To investigate potential subgroups of primary care–diagnosed patients with PMR based on self-reported pain and stiffness severity over time.

**Methods:**

A total of 652 people with an incident PMR diagnosis were recruited from English general practices and completed a baseline postal questionnaire. They were followed up with a further six questionnaires over a 2 year period. A total of 446 people completed the 2 year follow-up. Pain and stiffness were reported on a 0–10 numerical rating scale. Latent class growth analysis was used to estimate the joint trajectories of pain and stiffness over time. A combination of statistical and clinical considerations was used to choose the number of clusters. Characteristics of the classes were described.

**Results:**

Five clusters were identified. One cluster represented the profile of ‘classical’ PMR symptoms and one represented sustained symptoms that may not be PMR. The other three clusters displayed a partial recovery, a recovery followed by worsening and a slow, but sustained recovery. Those displaying classical PMR symptoms were in better overall health at diagnosis than the other groups.

**Conclusion:**

PMR is a heterogeneous condition, with a number of phenotypes. The spectrum of presentation, as well as varying responses to treatment, may be related to underlying health status at diagnosis. Future research should seek to stratify patients at diagnosis to identify those likely to have a poor recovery and in need of an alternative treatment pathway. Clinicians should be aware of the different experiences of patients and monitor symptoms closely, even where there is initial improvement.

**Rheumatology key messages**

- Pain and stiffness in polymyalgia rheumatica may be more variable across individuals than previously thought.
- In people with polymyalgia rheumatica, there are distinct groups with different symptom experiences over time.
- Future research into polymyalgia rheumatica should aim to identify those at risk of poor recovery.

## Introduction

PMR, a relatively common inflammatory rheumatological condition, is underresearched, especially in primary care, where the majority of patients are exclusively diagnosed and managed [1, 2]. PMR causes disabling pain and stiffness in the shoulder and hip girdles, often accompanied by elevated inflammatory markers (e.g. ESR, CRP), but it can present atypically or with non-specific symptoms, especially in the early stages. The mainstay of treatment is with oral glucocorticoids, which typically, although not always [3], bring about rapid relief of symptoms and improve physical function. Guidelines suggest a gradual tapering of glucocorticoid treatment over 18–24 months [4], although recent evidence suggests that treatment is often required for longer [5, 6], which may increase the likelihood of experiencing potentially serious treatment-related adverse effects.

To date, the majority of PMR research has been conducted in secondary care settings, which given the guidance on indications for referral for specialist review [7], potentially induces spectrum bias (i.e. study samples including patients with atypical presentation and/or more severe/difficult to treat disease). We therefore have little knowledge of the course of PMR in relation to its symptoms or treatment in the setting in which it is most frequently diagnosed and managed.

In order to provide an evidence base to understand the wider epidemiology of PMR, the PMR Cohort Study was established in 2012 [8, 9]. To our knowledge, this inception cohort of patients with PMR, recruited in England at the time of diagnosis, is the only prospective large-scale study of incident PMR in a primary care setting. A key aim of this cohort, supported by patient groups [10], is to better understand the prognosis of the condition. This may identify subgroups of patients who do not respond to glucocorticoids as expected or who are otherwise suitable for early interventions.

In this article we use latent class growth models [11] in data from the PMR Cohort Study to derive clinically recognizable groups of patients with differing patterns of pain and stiffness over 2 years. We consider whether the presentation of a patient at the time of diagnosis is different in those who go on to have different symptom trajectories.

## Methods

### Sample selection

Study procedures and the baseline sample have been described in detail elsewhere [8, 9]. Briefly, potential participants were identified when they were diagnosed with PMR by their general practitioner (GP) between June 2012 and June 2014. No study-specific diagnostic criteria were applied and participants were considered to have PMR if their assessing GP considered this to be their diagnosis and entered an associated Read code (the primary care clinical coding system used in the UK) into their medical record. Participating GPs were also provided with a copy of the British Society for Rheumatology (BSR) guidelines on the diagnosis of PMR to support making an accurate diagnosis [7]. Potential participants were mailed a baseline questionnaire. On return of the baseline questionnaire and consent to participate, participants received follow-up questionnaires 1, 4, 8, 12, 18 and 24 months after their initial diagnosis unless they explicitly withdrew consent or lost the capacity to continue (e.g. death, additional comorbidities).

### Patient and public involvement

The original idea for this study came from discussion with patients. Patients were involved in the design of data collection materials.

### Data collection

PMR-related pain and stiffness were reported on a 0–10 numerical rating scale (NRS) in each questionnaire. Participants also reported the duration of morning stiffness and their current prednisolone dose. Physical functioning was assessed using the Modified Health Assessment Questionnaire (mHAQ) [12, 13] on which normal functioning is considered to be a score of ≤0.33. General health was assessed using the European Quality of Life 5-Dimensions questionnaire (EQ-5D) [14], fatigue using the FACIT-Fatigue [15], insomnia using the Insomnia Severity Index [16], anxiety using the Generalized Anxiety Disorders-7 [17] and depression using the Patient Health Questionnaire-8 [18]. At each time point, participants were asked to shade in blank body manikins to indicate where they had pain and stiffness (separately). A transparent overlay was used to record areas of the body where pain/stiffness were reported. This method is widely used and has previously been shown to be reliable [19].

### Statistical analysis

Responders and non-responders to the 1 and 24 month questionnaires were compared on their sociodemographic, general health and PMR-specific characteristics at baseline using appropriate summary statistics. Similarly, the characteristics of the cohort over the seven time points were summarized appropriately.

#### Latent class growth analysis

Latent class growth analysis (LCGA) is a data-driven approach used to estimate the trajectory of a construct over time [11]. Rather than seeking to describe relationships among variables, LCGA identifies clusters of individuals within the dataset. In this case, LCGA was used to estimate the joint trajectories of pain and stiffness, as previous work has shown that patients consider them to be interlinked [20]. Due to the shape of average trajectories of pain and stiffness (Supplementary Fig. S1, available at *Rheumatology* online), which could not be well-represented by a polynomial curve, piecewise linear polynomial LCGA models were fitted to the data with a breakpoint at 1 month (time scale for analysis is months), i.e. separate polynomials were fitted between baseline and 1 month and between 1 and 24 months follow-up. Models were fitted using MPlus version 8.2 to ascertain the number of longitudinal clusters present [21]. Models were fitted to all available data, assuming data were missing at random. Parameters were estimated via maximum likelihood with robust standard errors, due to the non-normal distribution of responses. Each model was run with 5000 randomly generated starting values with 500 iterations in order to ensure the highest log-likelihood value was replicated.

Statistical and clinical considerations were taken into account when deciding on the number of clusters. Having assigned individuals to the cluster to which they had the highest probably of belonging, descriptive statistics were used to describe the characteristics of the clusters resulting from the LCGA model. Additional analyses were conducted in Stata 15.2 [22].

#### Sensitivity analyses

To test the robustness of our findings and missing data assumptions in terms of the shape of the identified trajectories, analyses were repeated in complete data, as in previous studies [23], and restricting the sample to those ≥50 years of age at diagnosis who reported morning stiffness duration ≥45 min at baseline, had bilateral pain and/or stiffness in the shoulders and were prescribed oral glucocorticoids at the time of diagnosis. This set of criteria was agreed upon by an independent group of rheumatologists to be clinically suggestive of PMR. In these analyses, we assumed that the appropriate number of clusters was the same as in the main analysis (for completeness, fitting models with up to seven clusters is shown in the Supplementary Materials, available at *Rheumatology* online) in order to compare the stability and shapes of the pain–stiffness trajectories over this number of clusters in these subsets of the data.

This study complies with the Declaration of Helsinki. Ethical approval for this study was received from the Staffordshire Research Ethics Committee (REC reference number 12/WM/0021) and all patients provided written informed consent.

## Results

### Cohort recruitment and retention

**Fig. 1:**
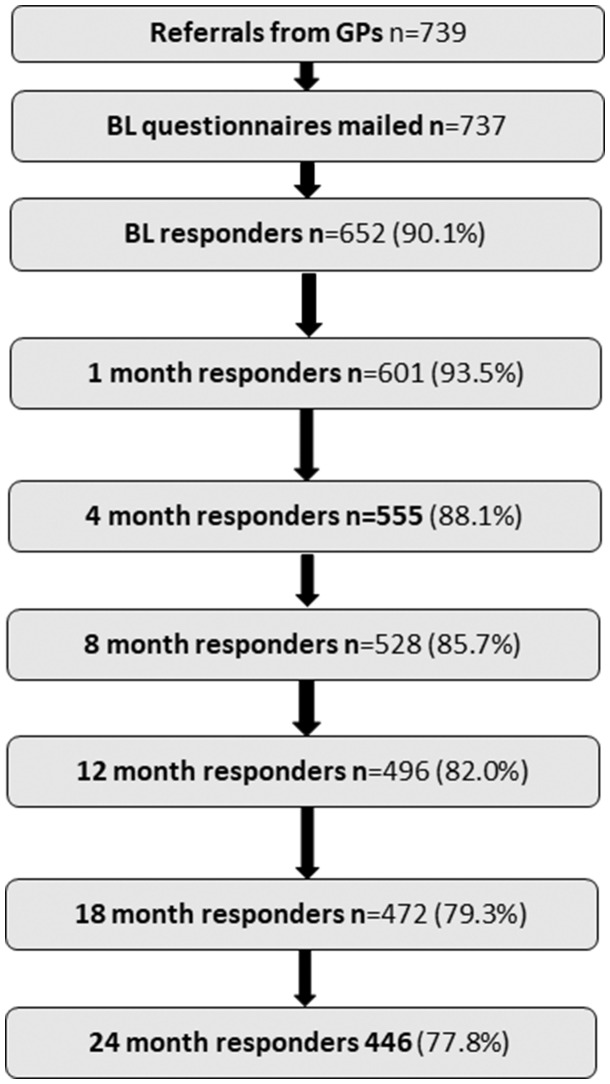
Study response and participation

Of those who were referred into the study by their GP (*n* = 739), 652 completed the baseline questionnaire and entered the cohort (adjusted response rate 90.1%). A total of 446 patients (77.8%) completed the 24 month questionnaire (Fig. 1). Younger age, female gender, lower occupational class, higher levels of pain, anxiety, depression and fatigue and poorer general health and physical functioning at baseline were associated with lower rates of subsequent response (Table 1).

**Table 1:** Patient characteristics and attrition at 1 and 24 months according to baseline characteristics

Baseline characteristics	1 month responders (*n* = 601)	1 month non-responders (*n* = 138)	24 month responders (*n* = 446)	24 month non-responders (*n* = 293)
Age, mean (s.d.), years	72.5 (9.3)	70.6 (9.0)	72.9 (8.7)	71.3 (10.3)
Gender, *n* (%)				
Male	232 (38.6)	15 (29.4)	185 (41.5)	62 (30.1)
Female	369 (61.4)	36 (70.6)	261 (58.5)	144 (69.9)
IMD rank, median (IQR)^a^	20 228 (12 849–25 248)	17 978 (10 045–25 443)	204 114 (13 566–25 395)	17 870 (9376–25 136)
Occupational class, *n* (%)				
Higher managerial, administrative and professional	140 (33.9)	4 (12.1)	121 (38.5)	23 (17.4)
Intermediate	116 (28.1)	9 (27.3)	82 (26.1)	43 (32.6)
Routine and manual	157 (38.0)	20 (60.6)	111 (36.4)	66 (50.0)
Ethnicity, *n* (%)				
White	589 (98.3)	49 (96.1)	438 (98.4)	200 (97.6)
Non-white	10 (1.7)	2 (3.9)	7 (1.6)	5 (2.4)
Employment status, *n* (%)				
Employed	70 (11.8)	6 (11.8)	45 (10.2)	31 (15.2)
Retired	476 (80.1)	36 (70.6)	363 (82.3)	149 (73.0)
Other	48 (8.1)	9 (17.7)	33 (7.5)	24 (11.8)
Marital status, *n* (%)				
Married	383 (64.1)	28 (56.0)	293 (66.0)	118 (57.8)
Widowed	133 (22.2)	10 (20.0)	91 (20.5)	52 (25.5)
Other	82 (13.7)	12 (24.0)	60 (13.5)	34 (16.7)
Pain (0–10 NRS), median (IQR)	8 (7–9)	8 (7–10)	8 (7–9)	8 (7–10)
Stiffness (0–10 NRS), median (IQR)	8 (7–9)	8 (6–10)	8 (7–9)	8 (6, 9)
Morning stiffness duration, *n* (%)				
≤60 min	167 (28.6)	17 (33.3)	119 (27.4)	65 (32.3)
>60 min	418 (71.5)	34 (66.7)	316 (72.6)	136 (67.7)
Currently taking prednisolone. *n* (%)				
No	17 (2.9)	2 (3.9)	6 (1.4)	13 (6.4)
Yes	576 (97.1)	49 (96.1)	436 (98.6)	189 (93.6)
PHQ-8 score, *n* (%)				
None/mild depression	439 (78.7)	31 (72.1)	341 (81.4)	129 (70.9)
Moderate/severe depression	119 (21.3)	12 (27.9)	78 (18.6)	53 (29.1)
GAD7 score, *n* (%)				
None/mild anxiety	493 (87.7)	37 (78.7)	378 (89.8)	152 (80.9)
Moderate/severe anxiety	69 (12.3)	10 (21.3)	43 (10.2)	36 (19.2)
mHAQ score, median (IQR)	0.375 (0–0.875)	0.75 (0.25–1.25)	0.375 (0–0.875)	0.5 (0.125–1.094)
EQ-5D score, median (IQR)	0.73 (0.59–0.85)	0.62, (0.415–0.69)	0.76 (0.62–0.88)	0.675 (0.52–0.8)
FACIT-Fatigue, mean (s.d.)	34.3 (12.4)	29.8 (11.3)	3.4 (11.7)	30.5 (13.1)
ISI score, *n* (%)				
No clinically significant/subthreshold insomnia	431 (76.4)	38 (76.0)	340 (79.8)	129 (68.6)
Moderate/severe clinical insomnia	133 (23.6)	12 (24.0)	86 (20.2)	59 (31.4)
Effect on intimate and sexual relationships, *n* (%)				
Not relevant	330 (56.0)	31 (63.3)	238 (54.3)	123 (61.5)
Little effect	154 (26.2)	10 (20.4)	127 (29.0)	37 (18.5)
Large effect	105 (17.8)	8 (16.3)	73 (16.7)	40 (20.0)

a Lower score is more deprived. IMD: Indices of Multiple Deprivation.

### PMR and general health characteristics of the sample over time

As previously reported [8], the median levels of pain and stiffness at diagnosis were 8 out of 10 (Table 2). For both symptoms, this fell to 2 out of 10 after the first month and remained low on average over the rest of the follow-up period, but varied greatly at the individual level (Supplementary Fig. S2, available at *Rheumatology* online). Similarly, 71% of people (*n* = 452) reported morning stiffness of more than 1 h at diagnosis, which fell to 26% at 1 month and remained at this level. Levels of physical functioning and fatigue also improved from diagnosis to 1 month before remaining stable. The proportion of people reporting anxiety, depression and insomnia decreased throughout the period of the study. General health, as measured by the EQ-5D remained stable throughout, as did self-reported BMI.

**Table 2:** Cohort characteristics over time

Characteristics	Baseline	Month 1	Month 4	Month 8	Month 12	Month 18	Month 24
Pain (0–10 NRS), median (IQR)	8 (7–9)	2 (1–4)	2 (1–4)	3 (1–5)	2 (1–4)	2 (1–5)	2 (0–5)
Stiffness (0–10 NRS), median (IQR)	8 (7–9)	2 (1–5)	3 (1–5)	3 (1–5)	3 (1–5)	3 (1–5)	3 (1–5)
Morning stiffness >60 min, *n* (%)	452 (71.1)	155 (26.4)	152 (26.4)	152 (27.7)	131 (27.0)	118 (26.0)	117 (27.0)
Report taking prednisolone, *n* (%)	625 (97.1)	564 (94.6)	518 (93.8)	463 (88.2)	397 (80.5)	323 (69.2)	255 (58.4)
Prednisolone dose, mg, mean (s.d.)	15.6 (7.4)	12.2 (6.6)	8.6 (5.1)	6.4 (4.1)	5.6 (3.8)	5.2 (4.2)	4.8 (3.4)
EQ-5D score, median (IQR)	0.73 (0.59–0.85)	NA	NA	NA	0.73 (0.62–0.81)	NA	0.73 (0.62–0.81)
mHAQ score, median (IQR)	0.402 (0–1)	0.25 (0–0.63)	0.25 (0–0.75)	0.25 (0–0.75)	0.25 (0–0.71)	0.25 (0–0.75)	0.25 (0–0.75)
FACIT-Fatigue score, mean (s.d.)	33.9 (12.3)	36.3 (12.2)	NA	NA	36.4 (11.2)	NA	36.8 (11.4)
Clinically relevant insomnia, *n* (%)	145 (23.6)	103 (17.7)	NA	NA	61 (12.8)	NA	56 (13.1)
Moderate/severe depression,^a^*n* (%)	131 (21.8)	85 (15.2)	NA	NA	58 (12.5)	NA	50 (12.4)
Moderate/severe anxiety,^b^*n* (%)	79 (13.0)	79 (13.7)	NA	NA	47 (10.0)	NA	36 (8.6)
BMI,^c^ mean (s.d.)	27.7 (5.4)	NA	NA	NA	27.9 (5.1)	NA	27.5 (5.0)

a Measured on the PHQ-8.

b Measured on the 7-item General Anxiety Disorder scale.

c BMI was restricted to values between 10 and 100 kg/m^2^.

IQR: interquartile range; NA: not measured.

### Pain and stiffness trajectories

**Fig. 2:**
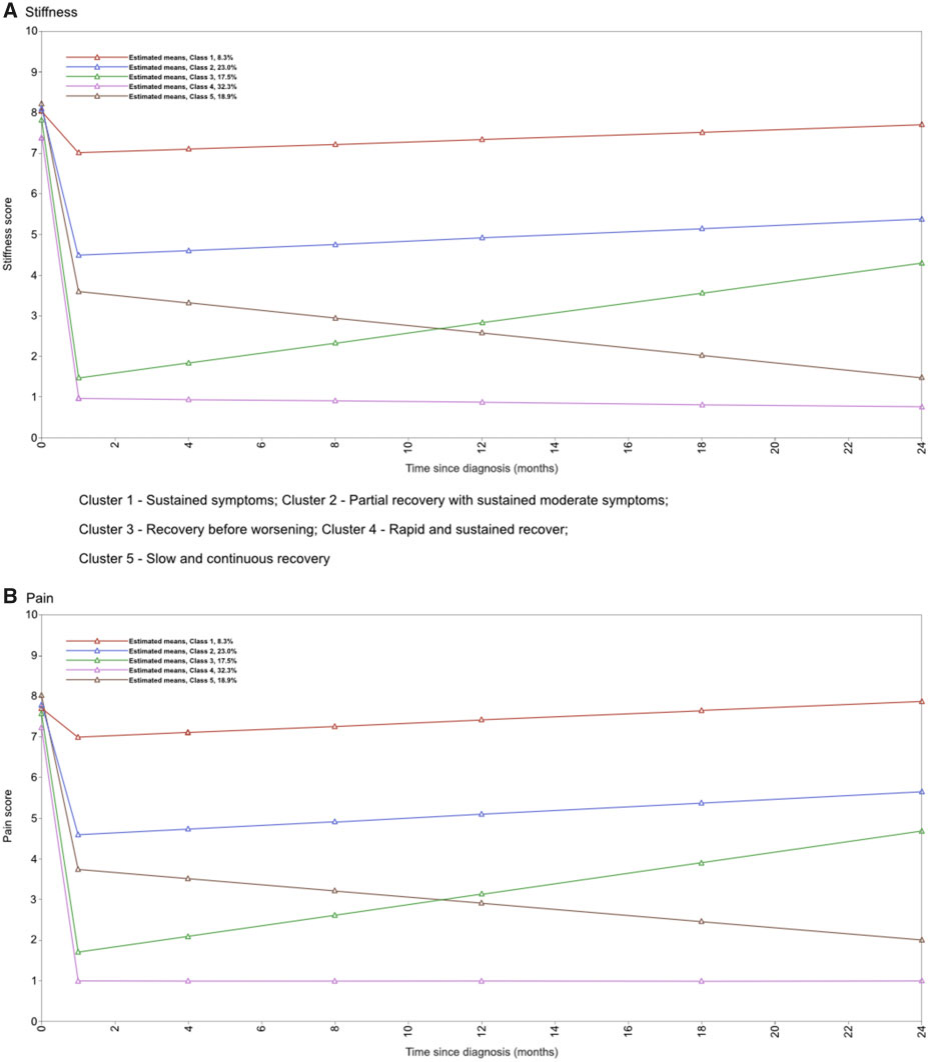
Fitted latent growth curves of pain and stiffness in the five-cluster model (*n* = 650)

All LCGA models converged. The model fit statistics [Akaike information criterion (AIC), Bayesian information criterion (BIC), sample-size adjusted BIC and parametric bootstrapped likelihood ratio test] suggested that six or more clusters would be the best fit, while the Vuong–Lo–Mendell–Rubin likelihood ratio test and Lo–Mendell–Rubin likelihood ratio test suggested only four clusters (Table 3). Considering the plots of pain and stiffness for four clusters, this model was deemed to be uninformative (Supplementary Fig. S3, available at *Rheumatology* online), as the trajectories were parallel and did not appear clinically meaningful. A model with six clusters was considered too complex. A five-cluster model was therefore deemed most appropriate (Fig. 2). This choice of model is broadly supported by the entropy statistic, cluster sizes and the average posterior probability of cluster membership. Clusters in this model could be assigned clinically useful meanings (outlined in Table 4, which describes the characteristics of the clusters at baseline). Variability of individual trajectories within each cluster can be seen in Supplementary Fig. S4, available at *Rheumatology* online.

**Table 3:** Latent class growth analysis—piecewise model selection (*n* = 650)^a^

Clusters	AIC	BIC	ABIC	Entropy	VLMR LRT	Adjusted LMR LRT	PB LRT	Cluster size	Average posterior probability of cluster membership
1	34 148	34 237	34 174	N/A	N/A	N/A	N/A	650	1.00
2	31 586	31 707	31 621	0.865	*P* < 0.0001	*P* < 0.0001	*P* < 0.0001	229, 421	0.90, 0.99
3	30 867	31 019	30 911	0.827	*P* = 0.003	*P* = 0.003	*P* < 0.001	129, 257, 264	0.90, 0.90, 0.94
4	30 605	30 789	30 659	0.808	*P* = 0.038	*P* = 0.041	*P* < 0.001	146, 219, 234, 51	0.84, 0.91, 0.89, 0.87
5	30 339	30 554	30 402	0.788	*P* = 0.063	*P* = 0.067	*P* < 0.001	52, 157, 106, 224, 111	0.87, 0.88, 0.78, 0.93, 0.75
6	30 185	30 431	30 257	0.793	*P* = 0.124	*P* = 0.129	*P* < 0.001	30, 117, 138, 106, 210, 49	0.71, 0.83, 0.82, 0.79, 0.92, 0.87
7	30 086	30363	30 166	0.786	*P* = 0.414	*P* = 0.418	*P* < 0.0001	208, 27, 130, 82, 109, 45, 49	0.94, 0.86, 0.79, 0.76, 0.85, 0.78, 0.68

a Two participants were excluded due to missing data (no pain or stiffness scores at any time point).

Significant *P*-value for VLMR LRT, adjusted LMR LRT and PB LRT (suggests model over model with one fewer cluster). The number in each cluster should be >5% of the sample size (i.e. >32). The average posterior probability of cluster membership should be >0.7.

ABIC: sample-size adjusted BIC; VLMR LRT: Vuong–Lo–Mendell–Rubin likelihood ratio test; LMR LRT: Lo–Mendell–Rubin likelihood ratio test; PB LRT: parametric bootstrapped likelihood ratio test. Model choice: choose the model with the lowest AIC, BIC, ABIC; entropy > 0.8.

**Table 4:** Baseline characteristics of the cohort by cluster (*n* = 650)

Characteristics	Cluster 1	Cluster 2	Cluster 3	Cluster 4	Cluster 5
	(*n* = 52)	(*n* = 157)	(*n* = 106)	(*n* = 224)	(*n* = 111)
Cluster description	Sustained symptoms	Partial recovery, sustained moderate symptoms	Recovery before worsening	Rapid and sustained recovery	Slow and continuous recovery
Age, mean (s.d. ), years	71.0 (10.0)	71.4 (10.5)	72.6 (9.0)	72.9 (8.0)	73.1 (9.5)
Female, *n* (%)	38 (73.1)	113 (72.0)	63 (59.4)	117 (52.2)	74 (66.7)
Pain (0–10 NRS), median (IQR)	8 (6.75–10)	9 (7–10)	8 (7–9.25)	8 (6–8)	8 (7–10)
Stiffness (0–10 NRS), median (IQR)	8 (6–9)	8 (7–10)	8 (7–9)	8 (6–8)	8 (7–10)
Morning stiffness >60 min, *n* (%)	33 (66.0)	114 (77.6)	78 (77.2)	148 (67.6)	78 (71.6)
Report taking prednisolone, *n* (%)	44 (86.3)	149 (96.1)	104 (98.1)	219 (98.7)	108 (100.0)
Prednisolone dose, mg, median (IQR)	15 (15–20)	15 (12.5–20)	15 (12–20)	15 (12.5–20)	15 (10–20)
EQ-5D score, median (IQR)	0.52 (0.19–0.69)	0.66 (0.52–0.76)	0.80 (0.70–1.0)	0.80 (0.71–1.0)	0.69 (0.62–0.80)
mHAQ score, median (IQR)	1.00 (0.50–1.25)	0.63 (0.38–1.13)	0.38 (0.00–0.75)	0.13 (0.00–0.50)	0.50 (0.25–1.00)
FACIT-Fatigue score, mean (s.d.)	23.2 (12.1)	30.0 (12.1)	36.1 (10.7)	39.0 (11.1)	32.3 (11.4)
Clinically relevant insomnia, *n* (%)	28 (58.3)	43 (28.7)	17 (17.0)	33 (15.8)	24 (22.9)
Moderate/severe depression, *n* (%)	17 (38.6)	29 (20.0)	10 (10.3)	10 (4.7)	13 (11.9)
Moderate/severe anxiety, *n* (%)	26 (56.5)	41 (28.3)	17 (17.7)	19 (9.2)	28 (26.2)
BMI, mean (s.d.)	28.4 (6.0)	29.3 (6.1)	27.5 (5.2)	26.9 (4.8)	26.9 (4.5)

IQR: interquartile range.

### Characteristics of individuals within the identified clusters

Baseline characteristics varied across the five clusters (Table 4). In general, those assigned to cluster 4 (rapid and sustained recovery) were in better health at baseline (considering scores on the EQ-5D and mHAQ, levels of depression, anxiety, fatigue and insomnia and BMI) and were more likely to be male. Those assigned to cluster 1 (sustained symptoms) were likely to be in poorer health and were less likely to be prescribed glucocorticoids.

Over the course of the study, the proportion of people prescribed glucocorticoids decreased in all clusters. The dose of prednisolone was similar across clusters in the first year, but after 12 months of follow-up the median dose was noticeably higher in cluster 1 than in the other clusters (Supplementary Table S1, available at *Rheumatology* online).

At the 2 year follow-up, 21% of people reported having been referred to a specialist for their PMR. This varied across clusters (Supplementary Table S2, available at *Rheumatology* online), from 13% in cluster 4% to 41% in cluster 1.

### Sensitivity analysis

#### Complete data

A total of 360 people had complete data for both the pain and stiffness scores at all seven time points. Missing data were due to a combination of non-response to the questionnaire and failure to complete the individual items. The LCGA model converged and a five-cluster solution was assumed, as described above. Full details of the model fitting process are presented in Supplementary Fig. S5 and Supplementary Table S3, available at *Rheumatology* online. Supplementary Figs S6 and S7, available at *Rheumatology* online show the final fitted trajectories and the individual variability within these, respectively. In those included in this analysis, 92% (*n* = 330) were allocated to the same cluster as in the original model (Supplementary Table S4, available at *Rheumatology* online). All of those with severe and sustained symptoms remained in this group and 95% of those with rapid and sustained recovery (classical PMR presentation) remained in this group. The major difference in the shape of the pain and stiffness trajectories was in the sustained symptoms cluster, which appeared to have more of a reduction in symptoms followed by an increase than in the original model.

#### PMR definition

A total of 453 patients (70%) met the stricter definition of PMR (Supplementary material, section ‘The tighter definition of PMR’, available at *Rheumatology* online); 24 people did not meet these criteria because they had not completed the items relating to either morning stiffness or glucocorticoid treatment. The majority of the remainder did not have a sufficiently long duration of morning stiffness [*n* = 128 (20%)]. The LCGA model converged, assuming a five-cluster solution. Full details of the model fitting process are presented in Supplementary Fig. S8 and Supplementary Table S5, available at *Rheumatology* online. The final fitted trajectories and the variability within them are shown in Supplementary Figs S9 and S10, respectively, available at *Rheumatology* online. Around 88% (*n* = 398) of people remained in the same cluster as in the original model. Where individuals moved between clusters, it tended to be between the clusters with poorer prognosis (Supplementary Table S6, available at *Rheumatology* online). More than 98% of those originally classified as having rapid and sustained recovery (classical PMR presentation) remained in this group. More than half of the sustained symptoms cluster of the original model were excluded from this sensitivity analysis. The shape of the pain and stiffness trajectories for the sustained symptoms group was slightly different in this sample compared with the full sample: the median pain score at baseline was higher than in the full sample before reducing to a lower level at 1 month and increasing again more rapidly after this time.

## Discussion

### Principal findings

This is the first large-scale study of PMR patients in primary care and reveals that there are multiple symptom trajectories. PMR, as diagnosed in primary care, appears to be a heterogeneous condition with patterns of symptom trajectories differing across patient groups. There is a group in which symptoms mirror the pattern of rapid and sustained recovery described in the literature. However, the majority of people report more varied and less straightforward symptom patterns.

Restricting the sample to those meeting criteria agreed upon by rheumatologists to be clinically suggestive of PMR excluded around one-third of the sample. The effect of these exclusions on the analysis was informative. Similar distinct groups were identified, however, the group with sustained symptoms was disproportionately affected. More than half of this group were excluded and the shape of the symptom trajectory also changed. A more substantial improvement in initial symptoms followed by a more extreme increase suggests that this group may not have had PMR, but had other conditions that were temporarily improved slightly by glucocorticoid treatment. Given the difficulty in making an accurate diagnosis of PMR [24], this scenario may not be uncommon and suggests that comorbidity may play a significant role in the accuracy of the diagnostic process and/or the symptom experience.

### Comparison of our cohort to current research literature

The starting dose of glucocorticoid was similar in this cohort to that described in a recent American study (15.6 *vs* 16.9 mg), as was the dose at 1 year (5.6 *vs* 5.9 mg) [5]. However, both studies show that treatment with glucocorticoids in PMR lasts considerably longer than suggested by current guidelines [4] and echoes some of the latest findings from large primary care database studies [6].

The causes of this longer glucocorticoid treatment require further study. Possible explanations include higher initial glucocorticoid dose, more severe baseline symptoms (e.g. levels of disability, inflammatory markers) and comorbidities. Shbeeb *et al.* [5] found no association between initial dose and time to permanent discontinuation of treatment, but did find an association between initial dose and time to maintaining doses <10 and <5 mg/day. Our finding that the median dose over time was higher in clusters with persistent pain and stiffness suggests that doctors may be maintaining higher doses to treat continuing symptoms rather than to ensure symptoms do not reoccur.

### Strengths and weaknesses

The major strength of this study compared with previous studies of PMR, is its recruitment from primary care, as <20% of patients with PMR ever see a rheumatology specialist [1] and only a subset of these will be referred at the time of diagnosis. By recruiting from general practices throughout England, we have included those who were diagnosed in both primary and secondary care settings, as specialists will refer patients back to primary care, where we would still have identified them for inclusion into the study. We were therefore able to avoid the spectrum bias potentially seen in studies recruiting exclusively from specialist settings. We ensured a high response rate at each follow-up point by keeping questionnaires short and using a reminder system for non-responders.

While the LCGA models can be estimated in the presence of missing data, we cannot be sure that data are missing at random, particularly as participants in poorer health at baseline were less likely to respond at follow-up. We chose to model pain and stiffness together in a dual-trajectory model due to the similarity in the shapes of the trajectories; we thereby included more information. This is in keeping with qualitative data that suggest that stiffness is intertwined with pain and also with function [20]. The same study also described a lack of consensus among patients regarding the best way to measure stiffness, with some considering the NRS chosen in our study to be appropriate and others preferring to relate stiffness to function. It could be argued that the 0–10 scale is a crude measure and may experience floor or ceiling effects. However, we do not consider there to be true floor and ceiling effects in this study, as participants were asked to consider 0 as ‘no pain’ and 10 as ‘pain as bad as can be’. Therefore scores of 0 and 10 should not be seen to ‘truncate’ the underlying pain/stiffness level of the participant, but represent no pain or the worst conceivable pain/stiffness.

Due to our recruitment strategy, we were reliant on the GP to accurately diagnose PMR. To ensure this, we provided all participating practices with a copy of the most recent BSR guidelines on the diagnosis of PMR [7]. To improve diagnostic accuracy further, we constructed a set of criteria based on clinical symptoms considered suggestive of PMR. While the clusters derived from our LCGA were similar after applying these criteria, ∼30% of people did not meet them. This may reflect inaccurate diagnosis in primary care, but may also reflect a difference in opinion between primary and secondary care on what constitutes PMR, especially in the context of multimorbidity. However, as we do not have data related to other morbidities, we were unable to relate the different pain and stiffness trajectories to the presence of other comorbidities (e.g. OA, GCA) or medications. The potential co-occurrence of GCA is a particular point to consider, as people with PMR are known to also have GCA in up to 20% of cases [25]. While it is unlikely that the presence of GCA has a dramatic effect on the reporting of pain and stiffness from PMR, it may influence glucocorticoid doses and hence symptom reporting.

### Implications for research and practice

We have successfully identified a group of people with a classical PMR presentation, where current treatment guidelines appear to be appropriate. However, we have also identified a group that may not have PMR and may require more robust diagnostic processes, potentially involving additional investigations, periods of close monitoring or early specialist referral for diagnostic clarification or alternative interventions. The logical next steps for research should be to develop processes to identify these two groups at an early stage. Attention should then be paid to those remaining, who display some response to glucocorticoid treatment but do not maintain a full reduction in symptoms in the long-term. Further studies need to address whether the use of adjunctive treatments (e.g. exercise) are of benefit. Future research should therefore consider how best to stratify patients at initial diagnosis to identify those with potential differential diagnoses or a need for a different treatment pathway (e.g. adjunctive physiotherapy, rheumatology management).

In the meantime, clinicians, especially those in primary care, should be aware of the potential for PMR to be a more heterogeneous condition than the literature suggests. In particular, they should consider alternative diagnoses in those who do not respond as expected to glucocorticoids in the first month and be aware of the potential for the return of symptoms or inability to taper their treatment effectively over time.

## Conclusions

It is unclear whether PMR is a single condition with multiple phenotypes or a group of conditions. It is unlikely that all of the heterogeneity seen in primary care is attributable to diagnostic inaccuracy. The spectrum of presentation, as well as varying responses to treatment, are made all the more difficult with the high level of multimorbidity in this group. The complex interaction of comorbidities and polypharmacy on the presentation and subsequent response to treatment is largely unknown in PMR but may be critical in our understanding of the condition. Future work should aim to delineate these subconditions early in the disease course in order for patients to receive appropriate management.

## Supplementary Material

kez533_Supplementary_Data

## References

[1] Barraclough K, Liddell WG, du Toit J et al Polymyalgia rheumatica in primary care: a cohort study of the diagnostic criteria and outcome. Fam Pract 2008;25:328–33. 18687983 10.1093/fampra/cmn044

[2] Helliwell T, Hider SL, Mallen CD. Polymyalgia rheumatica: diagnosis, prescribing, and monitoring in general practice. Br J Gen Pract 2013;63:e361–6. 23643235 10.3399/bjgp13X667231 PMC3635583

[3] Matteson EL, Maradit-Kremers H, Cimmino MA et al Patient-reported outcomes in polymyalgia rheumatica. J Rheumatol 2012;39:795–803. 22422492 10.3899/jrheum.110977

[4] Dejaco C, Singh YP, Perel P et al Current evidence for therapeutic interventions and prognostic factors in polymyalgia rheumatica: a systematic literature review informing the 2015 European League against Rheumatism/American College of Rheumatology recommendations for the management of polymyalgia rheumatica. Ann Rheum Dis 2015;74:1808–17. 26359489 10.1136/annrheumdis-2015-207578

[5] Shbeeb I, Challah D, Raheel S et al Comparable rates of glucocorticoid-associated adverse events in patients with polymyalgia rheumatica and comorbidities in the general population. Arthritis Care Res (Hoboken) 2018;70:643–7. 28704600 10.1002/acr.23320 PMC6475501

[6] Partington RJ, Muller S, Helliwell T et al Incidence, prevalence and treatment burden of polymyalgia rheumatica in the UK over two decades: a population-based study. Ann Rheum Dis 2018;77:1750–6. 30297332 10.1136/annrheumdis-2018-213883

[7] Dasgupta B, Borg FA, Hassan N et al BSR and BHPR guidelines for the management of polymyalgia rheumatica. Rheumatology (Oxford) 2010;49:186–90. 19910443 10.1093/rheumatology/kep303a

[8] Muller S, Hider S, Helliwell T et al The epidemiology of polymyalgia rheumatica in primary care: a research protocol. BMC Musculoskelet Disord 2012;13:102. 22703582 10.1186/1471-2474-13-102 PMC3406947

[9] Muller S, Hider SL, Helliwell T et al Characterising those with incident polymyalgia rheumatica in primary care: results from the PMR Cohort Study. Arthritis Res Ther 2016;18:200. 27605116 10.1186/s13075-016-1097-8 PMC5015343

[10] Muller S, O’Brien A, Helliwell T et al Support available for and perceived priorities of people with polymyalgia rheumatica and giant cell arteritis: results of the PMRGCAuk members’ survey 2017. Clin Rheumatol 2018;37:3411–8. 30066282 10.1007/s10067-018-4220-1

[11] Jung T, Wickrama K. An introduction to latent class growth analysis and growth mixture modeling. Soc Pers Psychol Compass 2008;2:302–17.

[12] Pincus T, Summey JA, Soraci SA Jr et al Assessment of patient satisfaction in activities of daily living using a modified Stanford Health Assessment Questionnaire. Arthritis Rheum 1983;26:1346–53. 6639693 10.1002/art.1780261107

[13] Kirwan JR, Reeback JS. Stanford Health Assessment Questionnaire modified to assess disability in British patients with rheumatoid arthritis. Br J Rheumatol 1986;25:206–9. 3708236 10.1093/rheumatology/25.2.206

[14] Euroqol Research Foundation [Homepage On The Internet]. Euroqol Research Foundation. 2016 [Cited December 2015]. http://Www.Euroqol.Org/ (July 2019, date last accessed).

[15] Webster K, Cella D, Yost K. The Functional Assessment of Chronic Illness Therapy (FACIT) Measurement System: properties, applications, and interpretation. Health Qual Life Outcomes 2003;1:79. 14678568 10.1186/1477-7525-1-79 PMC317391

[16] Morin CM. Insomnia: psychological assessment and management. New York: Guilford Press, 1993.

[17] Spitzer RL, Kroenke K, Williams JB et al A brief measure for assessing generalized anxiety disorder: the GAD-7. Arch Intern Med 2006;166:1092–7. 16717171 10.1001/archinte.166.10.1092

[18] Kroenke K, Strine TW, Spitzer RL et al The PHQ-8 as a measure of current depression in the general population. J Affect Disord 2009;114:163–73. 18752852 10.1016/j.jad.2008.06.026

[19] Lacey RJ, Lewis M, Jordan K et al Interrater reliability of scoring of pain drawings in a self-report health survey. Spine (Phila Pa 1976) 2005;30:E455–8. 16103839 10.1097/01.brs.0000174274.38485.ee

[20] Mackie SL, Hughes R, Walsh M et al “An impediment to living life”: why and how should we measure stiffness in polymyalgia rheumatica? PLoS One 2015;10:e0126758. 25955770 10.1371/journal.pone.0126758 PMC4425533

[21] Muthén LK, Muthén BO. Mplus user’s guide. 8th edn. Los Angeles, CA: Muthén & Muthén, 1998–2017.

[22] StataCorp. Stata statistical software: release 15. College Station, TX: StataCorp, 2017.

[23] Chen Y, Campbell P, Strauss VY et al Trajectories and predictors of the long-term course of low back pain: cohort study with 5-year follow-up. Pain 2018;159:252–60. 29112007 10.1097/j.pain.0000000000001097 PMC5771685

[24] Helliwell T, Muller S, Hider SL et al Challenges of diagnosing and managing polymyalgia rheumatica: a multi-methods study in UK general practice. Br J Gen Pract 2018;68:e783–e793. 30348883 10.3399/bjgp18X699557 PMC6193770

[25] Narváez J, Estrada P, López-Vives L et al Prevalence of ischemic complications in patients with giant cell arteritis presenting with apparently isolated polymyalgia rheumatica. Semin Arthritis Rheum 2015;45:328–33. 26186807 10.1016/j.semarthrit.2015.06.009

