# Supplementary material for: Longitudinal clusters of pain and stiffness in polymyalgia rheumatica: 2-year results from the PMR Cohort Study
Source: Rheumatology (Oxford). 2019 Nov 19;59(8):1906–15. doi: 10.1093/rheumatology/kez533 (PMC7382596; doi:10.1093/rheumatology/kez533)
Supplement: kez533_Supplementary_Data [file kez533_supplementary_data.docx]

**SUPPLEMENTARY MATERIAL**

**The tighter definition of PMR**

(as agreed by independent group of rheumatologists)

- aged 50 years or over at diagnosis
- morning stiffness duration to be ≥45 minutes at baseline
- bilateral pain and/or stiffness in the shoulders at baseline on body manikins
- prescribed oral glucocorticoids at the time of diagnosis

**Supplementary Table S1: Self-reported prednisolone dose (mg) (Median (IQR)) over time by Cluster**

| Cluster | Baseline | Month 1 | Month 4 | Month 8 | Month 12 | Month 18 | Month 24 |
| --- | --- | --- | --- | --- | --- | --- | --- |
| 1 Sustained symptoms | 15 (12.5, 20) | 10 (8.75, 15) | 10 (7.6, 14.5) | 6.25 (3, 11.25) | 9 (5, 15) | 10 (3.25, 15) | 5 (3.25, 6.75) |
| 2 Partial recovery, sustained moderate symptoms | 15 (12.5, 20) | 12.5 (10, 15) | 9 (7, 10) | 7 (5, 9) | 6 (4, 9) | 5 (4, 7.5) | 5 (3.5, 7.5) |
| 3 Recovery before worsening | 15 (11.5, 20) | 10.5 (10, 15) | 8 (6, 10) | 5.5 (4, 8) | 5 (2.5, 7) | 5 (3, 6) | 4 (3, 6) |
| 4 Rapid and sustained recovery | 15 (12.5, 20) | 10 (10, 15) | 7.5 (5, 9) | 5 (4, 7) | 5 (3, 6) | 4 (2, 6) | 3.25 (2, 6) |
| 5 Slow and continuous recovery | 15 (10, 20) | 10 (9, 14) | 8 (6, 10) | 6 (4, 8) | 5 (3, 6) | 4 (2, 5.75) | 4 (2, 5) |

IQR: interquartile range

**Supplementary Table S2: Proportion of individuals self-reporting being referred to a specialist for PMR in the 2-year follow-up questionnaire**

| Cluster | Referred, n (%) |
| --- | --- |
| 1 Sustained symptoms | 9 (40.9) |
| 2 Partial recovery, sustained moderate symptoms | 28 (28.9) |
| 3 Recovery before worsening | 19 (22.9) |
| 4 Rapid and sustained recovery | 21 (13.3) |
| 5 Slow and continuous recovery | 16 (18.6) |

**Supplementary Table S3. Latent Class Growth Analysis (LCGA) – piecewise model selection (n=360)**

| Cluster | AIC | BIC | ABIC | Entropy | VLMR LRT | Adjusted LMR LRT | PB LRT | Cluster sizes | Average posterior probability of cluster membership |
| --- | --- | --- | --- | --- | --- | --- | --- | --- | --- |
| 1 | 22887 | 22965 | 22901 | N/A | N/A | N/A | N/A | 360 | 1.00 |
| 2 | 21236 | 21340 | 32366 | 0.912 | 0.0014 | 0.0017 | <0.0001 | 217, 143 | 0.97, 0.98 |
| 3 | 20760 | 20892 | 20785 | 0.896 | 0.2179 | 0.2229 | <0.0001 | 145, 64, 151 | 0.96, 0.95, 0.95 |
| 4 | 20573 | 20732 | 20602 | 0.880 | 0.1857 | 0.1894 | <0.0001 | 57, 139, 93, 71 | 0.96, 0.97, 0.93, 0.87 |
| 5 | 20366 | 20552 | 20400 | 0.887 | 0.0586 | 0.0615 | <0.0001 | 72, 28, 120, 67, 73 | 0.93, 0.95, 0.97, 0.92, 0.87 |
| 6 | 20283 | 20497 | 20323 | 0.893 | 0.6032 | 0.6073 | <0.0001 | 28, 108, 25, 40, 68, 91 | 0.97, 0.95, 0.88, 0.86, 0.93, 0.91 |
| 7 | 20216 | 20457 | 20260 | 0.905 | 0.3633 | 0.3672 | <0.0001 | 90, 1, 107, 25, 68, 25, 44 | 0.89, 1.00, 0.96, 0.97, 0.93, 0.94, 0.88 |

Model choice: choose model with lowest AIC, BIC, ABIC; entropy>0.8. Significant p-value for VLMR LRT, adjusted LMR LRT and PB LRT (suggests model over model with one fewer clusters). Number in each cluster should be >5% of sample size (i.e.>18). Average posterior probability of cluster membership should be >0.7 *Note for this sensitivity analysis, a 5 cluster solution was assumed to compare to the main analysis. This table is for completeness only.* AIC: Akaike Information Criterion; BIC: Bayesian Information Criterion; ABIC: Sample-size adjusted BIC; VLMR LRT: Vuong-Lo-Mendell-Rubin likelihood ratio test; LMR LRT: Lo-Mendell-Rubin likelihood ratio test; PBLRT: parametric bootstrapped likelihood ratio test; p: p-value.

**Supplementary Table S4. Comparison of individual classifications in whole sample (n=652) and complete data (n=360)**

| n (%) |  | Cluster in whole sample (n=650) | | | | |
| --- | --- | --- | --- | --- | --- | --- |
|  |  | Severe sustained symptoms | Partial recovery, sustained moderate symptoms | Recovery before worsening | Rapid and sustained recovery | Slow and continuous recovery |
| Cluster in complete data (n=360) | Sustained symptoms | 14 (26.9) | 14 (8.9) | 0 | 0 | 0 |
|  | Partial recovery with sustained moderate symptoms | 0 | 66 (42.0) | 2 (1.89) | 0 | 4 (3.6) |
|  | Recovery before worsening | 0 | 0 | 67 (63.2) | 0 | 0 |
|  | Rapid and sustained recovery | 0 | 0 | 0 | 120 (53.6) | 0 |
|  | Slow and continuous recovery | 0 | 0 | 4 (3.8) | 6 (2.7) | 63 (56.8) |
|  | Excluded | 38 (73.1) | 77 (49.0) | 33 (31.1) | 98 (43.8) | 44 (39.6) |

**Supplementary Table S5. Latent Class Growth Analysis (LCGA) – piecewise model selection (n=453)**

| Cluster | AIC | BIC | ABIC | Entropy | VLMR LRT | Adjusted LMR LRT | PB LRT | Cluster sizes | Average posterior probability of cluster membership |
| --- | --- | --- | --- | --- | --- | --- | --- | --- | --- |
| 1 | 23996 | 24079 | 24015 | N/A | N/A | N/A | N/A | 453 | 1.00 |
| 2 | 22201 | 22313 | 22227 | 0.864 | P<0.0001 | P<0.0001 | P<0.0001 | 278, 175 | 0.97, 0.93 |
| 3 | 21719 | 21859 | 21751 | 0.834 | P=0.0276 | P=0.0293 | P<0.0001 | 82, 186, 185 | 0.88, 0.94, 0.92 |
| 4 | 21545 | 21714 | 21584 | 0.803 | P=0.1704 | P=0.1764 | P<0.0001 | 99, 156, 151, 47 | 0.85, 0.92, 0.87, 0.88 |
| 5 | 21361 | 21559 | 21406 | 0.796 | P=0.5323 | P=0.5374 | P<0.0001 | 62, 95, 89, 167, 40 | 0.74, 0.86, 0.82, 0.95, 0.87 |
| 6 | 21269 | 21495 | 21321 | 0.794 | P=0.4366 | P=0.4376 | P<0.0001 | 82, 145, 77, 58, 39, 52 | 0.86, 0.92, 0.76, 0.79, 0.88, 0.80 |
| 7 | 21170 | 21425 | 21228 | 0.815 | P=0.3432 | P=0.3446 | P<0.0001 | 58, 32, 146, 84, 95, 25, 13 | 0.80, 0.76, 0.93, 0.88, 0.82, 0.78, 0.89 |

Model choice: choose model with lowest AIC, BIC, ABIC; entropy>0.8. Significant p-value for VLMR LRT, adjusted LMR LRT and PB LRT (suggests model over model with one fewer clusters). Number in each cluster should be >5% of sample size (i.e.>23). Average posterior probability of cluster membership should be >0.7. Note for this sensitivity analysis, a 5 cluster solution was assumed to compare to the main analysis. This table is for completeness only AIC: Akaike Information Criterion; BIC: Bayesian Information Criterion; ABIC: Sample-size adjusted BIC; VLMR LRT: Vuong-Lo-Mendell-Rubin likelihood ratio test; LMR LRT: Lo-Mendell-Rubin likelihood ratio test; PBLRT: parametric bootstrapped likelihood ratio test; p: p-value.

**Supplementary Table S6. Comparison of individual classifications in whole sample and tighter PMR definition group (n=453)**

| n (%) |  | Cluster in whole sample (n=650) | | | | |
| --- | --- | --- | --- | --- | --- | --- |
|  |  | Sustained symptoms | Partial recovery with sustained moderate symptoms | Recovery before worsening | Rapid and sustained recovery | Slow and continuous recovery |
| Cluster in tighter PMR definition group sample (n=453) | Severe sustained symptoms | 22 (42.3) | 18 (11.5) | 0 (0.0) | 0 (0.0) | 0 (0.0) |
|  | Partial recovery with sustained moderate symptoms | 2 (3.9) | 86 (54.8) | 4 (3.8) | 0 (0.0) | 3 (2.7) |
|  | Recovery before worsening | 0 (0.0) | 0 (0.0) | 62 (58.5) | 0 (0.0) | 0 (0.0) |
|  | Rapid and sustained recovery | 0 (0.0) | 5 (3.2) | 2 (1.9) | 157 (70.1) | 3 (2.7) |
|  | Slow and continuous recovery | 0 (0.0) | 9 (5.7) | 7 (6.6) | 2 (0.9) | 71 (64.0) |
|  | Excluded | 28 (53.9) | 39 (24.8) | 31 (29.3) | 65 (29.0) | 34 (30.6) |

**Supplementary Figure S1. Distribution of pain and stiffness scores over 24 months**

**Supplementary Figure S2. Individual trajectories of (a) pain and (b) stiffness scores in 20 randomly selected individuals**

**Supplementary Figure S3. Fitted pain and stiffness trajectories for (a) pain and (b) stiffness with varying numbers of latent growth curves (n=650)**

**Supplementary Figure S4: Individual trajectories of (a) pain and (b) stiffness within the five clusters of the chosen model (n=650)**

**Supplementary Figure S5: Fitted (a) pain and (b) stiffness trajectories with varying numbers of clusters fitted (n=360)**

**Supplementary Figure S6. Fitted latent growth curves of pain and stiffness in 5 cluster model (n=453) a)** Pain; **b)** Stiffness. Cluster 1 - Partial recovery with sustained moderate symptoms; Cluster 2 - Sustained symptoms Cluster 3 - Rapid and sustained recovery; Cluster 4 - Recovery before worsening; Cluster 5 - Slow and continuous recovery.

**Supplementary Figure S7: Individual trajectories of pain and stiffness within the five clusters (n=360)**

**Supplementary Figure S8. Fitted pain and stiffness trajectories with varying numbers of clusters fitted (n=453)** (a) pain; (b) stiffness. Note for this sensitivity analysis, a 5 cluster solution was assumed to compare to the main analysis. This figure is for completeness only.

**Supplementary Figure S9. Fitted latent growth curves of pain and stiffness in 5 cluster model (n=453)** a) Pain; b) stiffness. Cluster 1 - Recovery before worsening; Cluster 2 - Partial recovery with sustained moderate symptoms; Cluster 3 - Slow and continuous recovery; Cluster 4 - Rapid and sustained recovery; Cluster 5 - Sustained symptoms

**Supplementary Figure S10. Individual trajectories of pain and stiffness within the five clusters (n=453)**
